# Distribution of cytochrome P450 2C, 2E1, 3A4, and 3A5 in human colon mucosa

**DOI:** 10.1186/1472-6904-5-4

**Published:** 2005-10-27

**Authors:** Ina Bergheim, Christiane Bode, Alexandr Parlesak

**Affiliations:** 1 Hohenheim University (140), Dep. Physiology of Nutrition, Stuttgart, Germany; 2 Department of Pharmacology and Toxicology, University of Louisville Health Sciences Center, Louisville, KY, USA

## Abstract

**Background:**

Despite the fact that the alimentary tract is part of the body's first line of defense against orally ingested xenobiotica, little is known about the distribution and expression of cytochrome P450 (CYP) enzymes in human colon. Therefore, expression and protein levels of four representative CYPs (CYP2C(8), CYP2E1, CYP3A4, and CYP3A5) were determined in human colon mucosa biopsies obtained from ascending, descending and sigmoid colon.

**Methods:**

Expression of CYP2C, CYP2E1, CYP3A4, and CYP3A5 mRNA in colon mucosa was determined by RT-PCR. Protein concentration of CYPs was determined using Western blot methods.

**Results:**

Extensive interindividual variability was found for the expression of most of the genes. However, expression of CYP2C mRNA levels were significantly higher in the ascending colon than in the sigmoid colon. In contrast, mRNA levels of CYP2E1 and CYP3A5 were significantly lower in the ascending colon in comparison to the descending and sigmoid colon. In sigmoid colon protein levels of CYP2C8 were significantly higher by ~73% than in the descending colon. In contrast, protein concentration of CYP2E1 was significantly lower by ~81% in the sigmoid colon in comparison to the descending colon.

**Conclusion:**

The current data suggest that the expression of CYP2C, CYP2E1, and CYP3A5 varies in different parts of the colon.

## Background

Throughout the last decades drug metabolism in the alimentary tract has received growing attention as part of the body's first line of defense against orally ingested harmful xenobiotica. Most xenobiotic compounds require an enzymatic activation to form a carcinogen or toxicant. The reactive intermediates resulting form enzymatic drug metabolism are often unstable and therefore are unlikely to be transported from the liver to other tissues to exert toxicity. Therefore, the chemical toxicity found in extrahepatic tissue often results from cellular metabolic activities in the organ. However, knowledge on the variability and regulation of expression of drug metabolizing enzymes in the human gastrointestinal tract and particularly the large intestine is poor in comparison with the "classical" drug metabolizing organs (e.g. liver).

Cytochrome P450 (CYP) is a multi-gene superfamily of heme-containing enzymes catalyzing the oxidative metabolism of many compounds [1]. CYP families 1, 2, and 3, which are the main CYP families participating in the metabolism of xenobiotics, are highly expressed within the liver, but are also expressed in extrahepatic tissues (for review see [2]). Members of the CYP families 2 and 3, and herein especially CYP2C and CYP3A4, are present in relative high concentrations in small intestinal epithelium [3-5], and it has been suggested that they facilitate a barrier function to protects the small intestine from toxic xenobiotics [4]. Furthermore, it has been shown that total CYP content increases slightly in the progression from duodenum to jejunum and subsequently decreases significantly in the ileum [3]. Despite the fact that it has been suggested that the absence of some of these microsomal enzymes in the colon may be involved in the comparably high incidence of carcinoma in this organ [4], information available on the expression of CYPs in the large intestine of humans is limited and some of the available data are contradictory. Therefore, the main purpose of the present study was to evaluate the expression pattern, protein concentration, and distribution of four representative CYPs (CYP2C, CYP2E1, CYP3A4, and CYP3A5) in ascending, descending and sigmoid human colon mucosa of virtually healthy subjects. Furthermore, protein levels were related to mRNA expression pattern.

## Material and methods

### Subjects

The study was approved by the Ethics Committee of the Medical Association Stuttgart, Germany. Informed consent was obtained from all subjects included in the study. During routinely performed coloscopies, two biopsy specimens (each 5–10 mg) of normal colon mucosa were obtained from either ascending (n = 10), descending (n = 7), or sigmoid (n = 24) colon of a total of 41 volunteers (age 30 – 80). None of the subjects displayed any macroscopic evidence of colonic neoplasia or other disease in colon. All patients completed a questionnaire concerning factors that may influence the expression of cytochrome P450 such as medication, smoking, and alcohol consumption (see Table 1). Neither anthropometrics nor lifestyle and drug intake differed significantly between donors of biopsy specimens of ascending, descending, and sigmoid colon. Furthermore, no significant correlation was observed between these parameters and the protein levels as well as mRNA expression patterns of CYPs investigated. Subjects and controls did not consume drugs known to interfere with or to induce CYPs investigated in this study.

**Table 1:** Anthropometic and life style data of subjects.

**Parameter**	**Ascending Colon**	**Descending Colon**	**Sigmoid Colon**
**n**	10	7	24
**Age**	52 ± 9	54 ± 15	55 ± 9
**Sex : Female/male**	5/5	1/6	8/16
**BMI**	26.5 ± 5.7	27.8 ± 4.9	25.4 ± 4.0
**Cigarette use**			
**Yes/No**	3/7	3/4	4/20
**(number/d)**	2.8 ± 6.2	8.3 ± 9.8	1.5 ± 4.6
**Alcohol consumption**			
**Yes/No**	3/7	2/5	12/12
**(g/d)**	9.4 ± 14.6	8.2 ± 7.8	9.8 ± 12.6

All data are expressed as means ± SD. (BMI = body mass index)

### Tissues and isolation of total RNA and protein

All colon mucosa specimens were immediately frozen in liquid nitrogen after excision and stored at -80°C. Both total RNA and protein was isolated using Trizol reagent (Invitrogene, Gaithersburg, MD, USA).

### Electrophoresis and immunoblotting

Protein concentration was determined using a commercially available Bradford assay (BioRad, Munich, Germany). Twenty to 30 μg of total protein were separated by electrophoresis through a 9% SDS-polyacrylamid gel and was transferred onto a nitrocellulose membrane. To ensure equal loading of samples, membranes were stained with Ponceau red. In addition, some blots were probed for β-actin (Sigma, Munich, Germany) to ascertain identical protein loading of samples. Membranes were blocked in 5% non-fat milk in Tris-buffered saline-Tween 20 (TBST, 0.01% v/v Tween 20) and probed with dilutions of primary antibodies in TBS followed by an incubation with the secondary antibody. Anti-CYP2E1 and anti-CYP3A4 antibodies were generous gifts of Dr. M. Ingelman-Sundberg, Karolinska Institute, Stockholm, Sweden; anti-CYP2C8 and anti-CYP3A5 were purchased from Chemicon, Inc. (Frankfurt, Germany). The protein/antibody complex was visualized by enhanced chemiluminescence (SuperSignal^® ^West Dura, Pierce, Bad Godesberg, Germany). Blots were photographed (Camera LAS 1000, Fuji, USA) and densitometric analysis was performed using the software AIDA (Raytest, Isotopenmessgeraete, Straubenhardt, Germany). As positive control and for semiquantification, serial dilutions of microsomes and Supersomes derived from cell lines and clones expressing human CYP2C8, CYP2E1, CYP3A4, and CYP3A5 (Gentest Corporation, Woburn, MA, USA) respectively were loaded on each gel.

### Reverse transcription and PCR

The integrity and concentration of RNA was analyzed in a 1.2% agarose gel. First-strand complementary DNA was synthesized from 200 ng of total RNA using a First-Strand cDNA Synthesis Kit (Invitrogen, Gaithersburg, MD, USA). Sequences of primers are summarized Table 2. The PCR reaction consisted of 0.6 μl of cDNA, 10 × PCR buffer, 200 μM dNTPs (Boehringer, Mannheim, Germany), BSA (0.25 mg/ml), DMSO (2% v/v), 0.5 μM of specific primer and 0.5 U Taq-polymerase (Promega, Madison, WI, USA), and water to a final volume of 10 μl. For amplifications of the four cytochrome P450 cDNAs, PCR-conditions were as follows: 3 s at 94°C, 3 s at 45°C, 30 s at 72°C, for 32 cycles. Amplification of histone 3.3 was performed applying the following conditions: 3 s at 94°C, 3 s at 45°C, and 30 s at 72°C for 30 cycles. All PCR amplifications were carried out in triplicate in a Rapid Cycler (Idaho Tec., USA) within the linear range of the reaction. PCR products were separated in a 1.5% agarose gel, stained with ethidium bromide and photographed using a digital camera from Biometra (Goettingen, Germany). To ensure the success of PCR, human liver cDNA was used as a positive control.

**Table 2:** Primers used for RT-PCR analysis

	**Sense primer location**	**Antisense primer location**	**PCR product (bp)**	**Reference**
**Histone 3.3**	GCGTGCTAGCTGGATGTCTT	CCACTGAACTTCTGATTCGC	150	[18]
**CYP2C8-19**	GCTAAAGTCCAGGAAGAGATTGA	TCCTGCTGAGAAAGGCATGAAGT	332	[19]
**CYP2E1**	AGCACAACTCTGAGATATGG	ATAGTCACTGTACTTGAACT	365	[19]
**CYP3A4**	CCAAGCTATGCTCTTCACCG	TCAGGCTCCACTTACGGTGC	324	[20]
**CYP3A5**	TGTCCAGCAGAAACTGCAAA	TTGAAGAAGTCCTTGCGTGTC	472	[20]

### Statistical analysis

Mann-Whitney U test, Chi-square-test for crosstabulation tables, and analysis of variances *(ANOVA) *with the Posthoc test of Tukey was used for the determination of statistical significance as appropriate. A *P *value of less than 0.05 was selected as the level of significance before the study.

## Results

### Expression of CYP2C, CYP2E1, CYP3A4, and CYP3A5 mRNA in ascending, descending, and sigmoid colon

**Figure 1:**
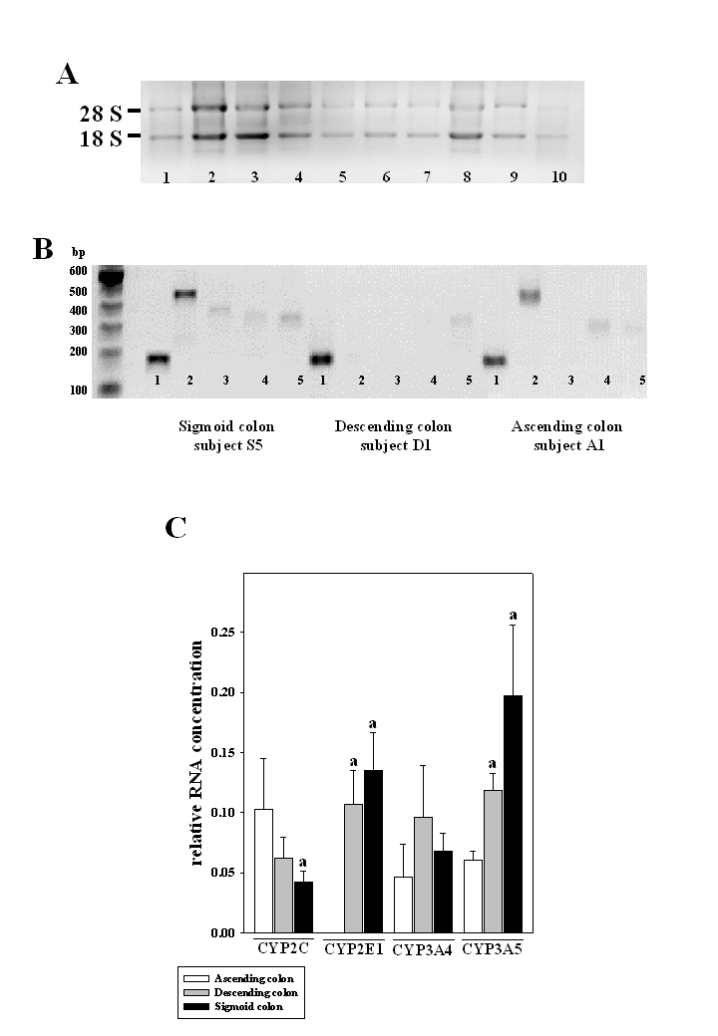
**CYP3A5, CYP2E1, CYP3A4, and CYP2C mRNA expression in human colon mucosa obtained ascending, descending, and sigmoid colon**. (A) Representative agarose gel of mRNA integrity. Lane 1 and 10 = positive control, lane 2–9 = RNA extracted from colonic mucosa biopsies. (B) Representative photomicrograph of RT-PCR products of three subjects. All measurements were carried out in triplicate. Lane 1 = Histone 3.3; Lane 2 = CYP3A5; Lane 3 = CYP2E1; Lane 4 = CYP3A4; Lane 5 = CYP2C, bp = base pairs, A = ascending colon, D = descending colon, S = sigmoid colon. (C) Quantitative analysis of mRNA expression of CYP2C, CYP2E1, CYP3A4, and CYP3A5 in human ascending, descending, and sigmoid colon. Data are normalized to histone 3.3 expression. Data are means ± SEM. (n.d. = not detectable, ^a^*P *< 0.05 compared to ascending colon)

The presence of a band of the correct size in agarose gels was regarded as evidence of gene expression. Expression of the housekeeping gene histone 3.3 was detected in all samples. A total of 10 specimens obtained from the ascending colon, 6 specimens from the descending colon and 21 specimens obtained form the sigmoid colon were included in the analysis of mRNA expression. Three RNA samples obtained form the sigmoid colon had to be excluded as RNA concentrations were too low. Representative gels depicting RNA integrity and results of RT-PCR measurements are shown in Figure 1.

Expression of CYP2C was found to be higher in the ascending colon than in the descending and sigmoid colon. However, due to large interindividual variability differences in CYP2C expression were only significant between the ascending and the sigmoid colon. In the ascending colon expression of CYP2E1 was not detectable. In the descending and the sigmoid colon expression of CYP2E1 did not differ significantly, however expression of CYP2E1 was detectable. Expression of CYP3A4 did not differ between the three regions of colon investigated. In contrast, CYP3A5 mRNA expression was significantly higher in the descending and the sigmoid colon in comparison to the ascending colon. Specifically, in the descending colon CYP3A5 mRNA expression was ~2-fold and in the sigmoid colon ~3-fold higher than in the ascending colon. No differences in CYP3A5 expression were found between the descending and sigmoid colon.

### Protein levels of CYP2C8, CYP2E1, CYP3A4, and CP3A5 in the descending and sigmoid colon

**Figure 2:**
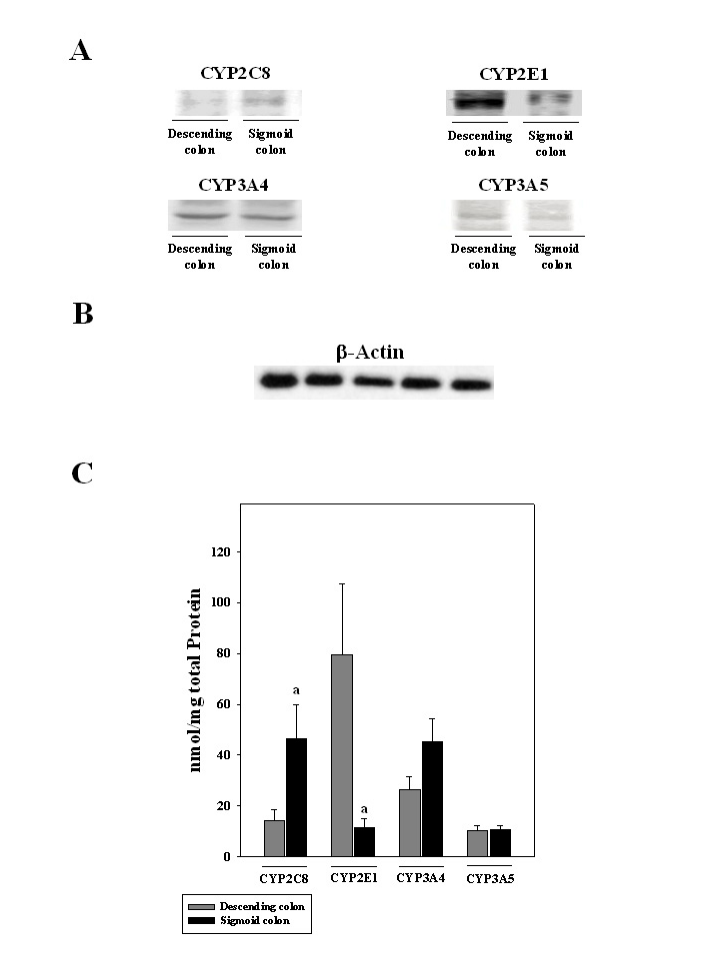
**CYP2E1, CYP2C8, CYP3A4, CYP3A5 protein levels in normal human colon mucosa obtained from descending and sigmoid colon**. (A) Representative Western blot of CYP2E1, CYP2C8, CYP3A4, CYP3A5. (B) Representative Western blot of β-actin performed in 5 different individuals. (C) Quantitative analysis of blots. Data are means ± SEM. (^a^*P *< 0.05 compared to descending colon)

Due to a low protein content of some mucosal biopsies, Western blot analyses of CYP2E1, CYP3A4, and CYP3A5 were only performed in 6 tissue specimens obtained from the descending and 24 from sigmoid colon. Since higher protein concentrations were needed for the detection of CYP2C8, protein concentration of CYP2C8 was only determined in 17 specimens taken from sigmoid colon. Biopsies obtained from the ascending region of the colon were not included in the analysis as sufficient protein concentrations for Western blot analysis were only obtained from four samples. Figure 2 depicts representative Western blot and quantitative analysis of protein.

The mean protein level of CYP2C8 was significantly lower in the descending colon when compared with the sigmoid colon. Specifically, protein levels of CYP2C8 were found to be ~73% lower in the descending colon in comparison to the sigmoid colon. In contrast, protein concentration of CYP2E1 was significantly higher by ~81% in the descending colon compared to the sigmoid colon. However, no significant differences were found when comparing protein levels of CYP3A4 and CYP3A5 between the descending and sigmoid colon.

### Relation of protein levels and mRNA expression pattern of CYPs in sigmoid colon

**Figure 3:**
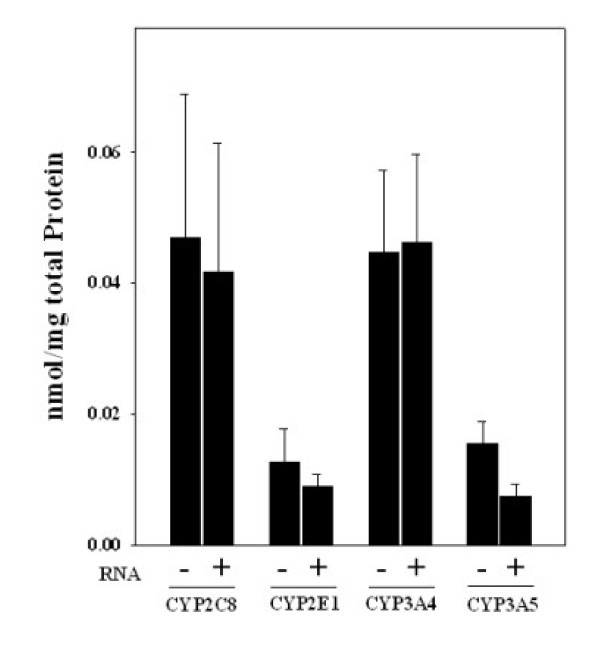
**Relation of CYP2E1, CYP2C8, CYP3A4, CYP3A5 protein levels and mRNA expression pattern in normal human colon mucosa obtained from sigmoid colon**. Comparison of protein levels of CYP2E1, CYP2C8, CYP3A4, and CYP3A5 of subjects with detectable mRNA expression and subjects with no detectable mRNA expression of the respective CYP. Data are means ± SEM.

To address the question whether mRNA profiles correlated CYP protein concentration in colonic mucosa, protein levels of CYPs of subjects with detectable mRNA expression were compared with those with undetectable mRNA expression of the respective CYP. Since the sample number of biopsies obtained from the ascending and descending colon was too small to perform a statistical analysis (ascending: n = 4; descending: n = 6), this comparison was only performed for the sigmoid colon. Results are summarized in Figure 3. Interestingly, no significant differences were found when comparing protein levels of CYP2C8, CYP2E1, CYP3A4, and CYP3A5 of subjects with detectable mRNA expression of these CYPs with those with undetectable mRNA levels. No correlation was found between CYP2E1 expression and individual alcohol consumption.

## Discussion

### Abundance of CYPs differs between ascending, descending, and sigmoid colon and between individuals

It has been suggested that the expression of CYPs (e.g. the absence of expression of certain CYPs) in the colon might be an important factor in the susceptibility of this organ for cancer. Expression of members of the CYP2C-, CYP2E-, and CYP3A-family in normal mucosa along with substantial interindividual differences in the expression levels of CYPs in the colon has been reported by others [6-11]. However, some of the available data are contradictory and most studies either determined mRNA or protein levels. Furthermore, detailed information on the distribution of CYPs within the colon (e.g. distal vs. proximal colon) is lacking. For example, McKay et al. [6] detected CYP3A protein in two out of 13 morphologically normal colon mucosa specimens of patients with neoplasia in the colon. Similar results were also reported by Mercurio et al.[7] and McKinnon et al.[8] for CYP3A4 and CYP3A5 mRNA expression in colon. Using immunohistochemical methods, Yokose et al. [9] reported the presence of CYP2C(8–19) in healthy human colon mucosa also finding large interindividual differences. In contrast, Western blot analyses by de Waziers et al. [10] and Massaad et al. [11] applying conventional immunoperoxidase staining procedure failed to detect the expression of cytochromes P450 2C8-10 and 2E1 protein in human colon mucosa. In the present study, the expression and protein levels of four representative CYPs were determined in different regions of the large intestine. At the levels of mRNA expression CYP2C, CYP2E1, and CYP3A5 concentration was found to be significantly different between the three different regions investigated with CYP2C mRNA expression being significantly higher in proximal regions of the colon than in the distal (e.g. descending and sigmoid colon). In contrast, mRNA expression of CYP2E1 and CYP3A5 was significantly lower in the proximal colon (i.e. ascending) than in the distal part of the organ. However, in accordance with the findings of others, mRNA expression of all four CYPs was found to vary extensively between individuals, even though expression of histone 3.3, which was used as housekeeping gene, was detected in all samples. At the level of protein, CYP2C8 concentration was found to be lower in the descending colon when compared with the more distal sigmoid region of the colon. Contrary, CYP2E1 protein levels were higher in descending colon than in the sigmoid colon. Taken together, these data suggest that CYP expression in colon not only varies among individuals but also among different regions of the organ.

### CYP protein levels and mRNA expression are not related

Studies of the mRNA and protein expression of CYP3A4 and CYP3A5 in rat duodenum and kidney, as well as the expression of CYP2C7 (corresponding to human CYP2C8) and CYP2E1 in rat colon mucosa, revealed a dissociation of mRNA expression and protein levels of CYPs. [12] Hakkak et al. [13] suggested that the expression of CYPs is not solely regulated at the transcriptional level. This is supported by results of *in vitro *investigations in rat hepatocytes, which indicated that protein level of CYP2E1 is regulated by posttranscriptional ligand-dependent stabilization of the enzyme [14]. Similar mechanisms have been described for rat and human CYP3A [4,15,16]. Furthermore, *in vitro *studies using cultured hepatocytes showed that only ~60–70% of mRNA encoding for CYP2E1 is translated [17]. Indeed, in the present study, no differences with respect to protein levels of subjects with detectable and undetectable mRNA expression of the CYPs were found.

## Conclusion

In summary, interindividual variability seems to be a characteristic of CYP expression in colon as has been reported by others before [6-11]. However, in the present study we found significant differences of CYP2C(8), CYP2E1 and CYP3A5 mRNA expression and protein levels between different regions of the colon (e.g. ascending, descending, and sigmoid colon). Metabolic implications of this "zonification" remain to be determined. Nevertheless, differences found in the present study might result in alterations of detoxification of carcinogens or pro-carcinogens and therefore contribute to high susceptibility of this organ to carcinoma.

## Competing interests

The author(s) declare that they have no competing interests.

## Authors' contributions

IB has made substantial contributions to acquisition of data, the biochemical analysis, and the drafting of article. CB has made substantial contribution to conception and design as well as the interpretation of data. AP has been involved in the design, the drafting of the article, and revised it critically for intellectual content. All authors have given final approval of the version to be published.

## Pre-publication history

The pre-publication history for this paper can be accessed here:


